# P-319. Discordance Between Objective and Perceived Risk for Contracting HIV and Association with PrEP Engagement in Transgender Individuals

**DOI:** 10.1093/ofid/ofaf695.538

**Published:** 2026-01-11

**Authors:** Meghan F Anderson, Meredith Zoltick, Rahwa Eyasu, Emade Ebah, Phyllis Bijole, Miriam Jones, Dorcas Salifu, Habib Omari, Ashley Davis, Sarah Kattakuzhy, Elana S Rosenthal

**Affiliations:** University of Maryland, School of Medicine, Annandale, VA; University of Maryland Baltimore - Institute of Human Virology, Baltimore, Maryland; Institute for Human Virology IHV, University of Maryland School of Medicine, Washington, District of Columbia; Institute for Human Virology IHV, University of Maryland School of Medicine, Washington, District of Columbia; HIPS.org, Washington, District of Columbia; HIPS.org, Washington, District of Columbia; University of Maryland Baltimore - Institute of Human Virology, Baltimore, Maryland; University of Maryland Baltimore, Baltimore, Maryland; University of Maryland, Baltimore, Washington, District of Columbia; Institute for Human Virology IHV, University of Maryland School of Medicine, Washington, District of Columbia; Institute for Human Virology IHV, University of Maryland School of Medicine, Washington, District of Columbia

## Abstract

**Background:**

Transgender individuals are at high risk for contracting HIV but have low rates of PrEP use. We aimed to understand the concordance between self-perceived HIV risk and CDC PrEP indications to identify ways to improve PrEP engagement in transgender individuals.

**Table 1:**
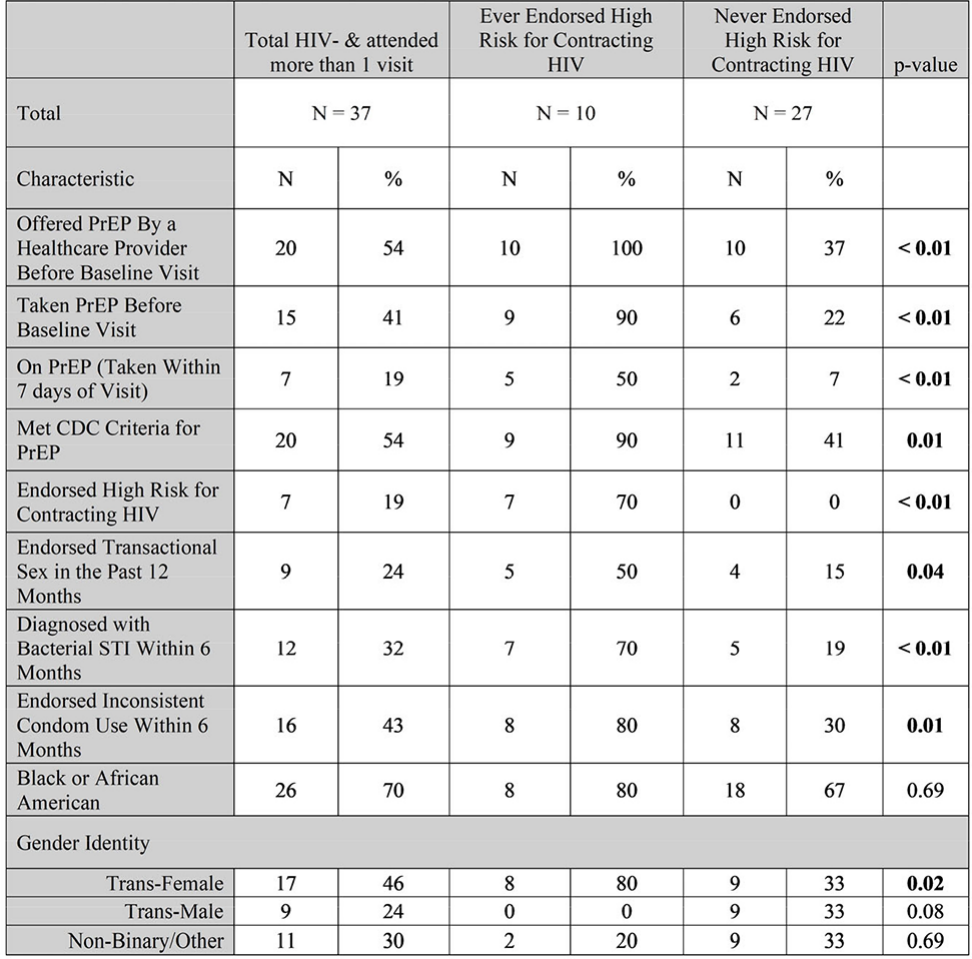
Baseline Characteristics of HIV Negative Participants by Endorsement of High Risk for Contracting HIV

**Table 2:**
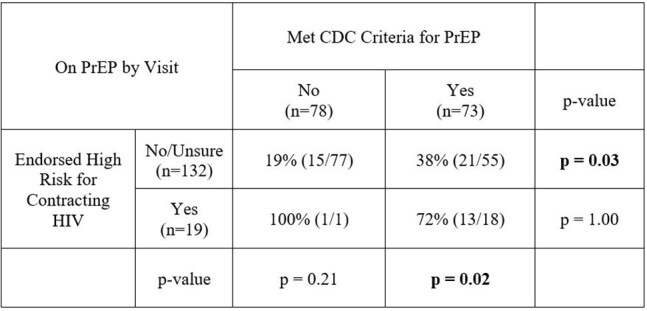
Visit Specific Rates of PrEP Engagement Among HIV Negative Participants by CDC Criteria and Endorsement of High-Risk

**Methods:**

PATCH was a longitudinal natural history study of transgender individuals in Washington, DC. Participants completed laboratory testing and surveys assessing HIV risk behaviors and PrEP status every 3 months for 1 year. The cohort was categorized into 2 groups: ever endorsed high-risk for contracting HIV (EE) or never endorsed (NE).

Fisher’s exact test and Cohen’s Kappa statistic were used for statistical analysis.

**Figure 1:**
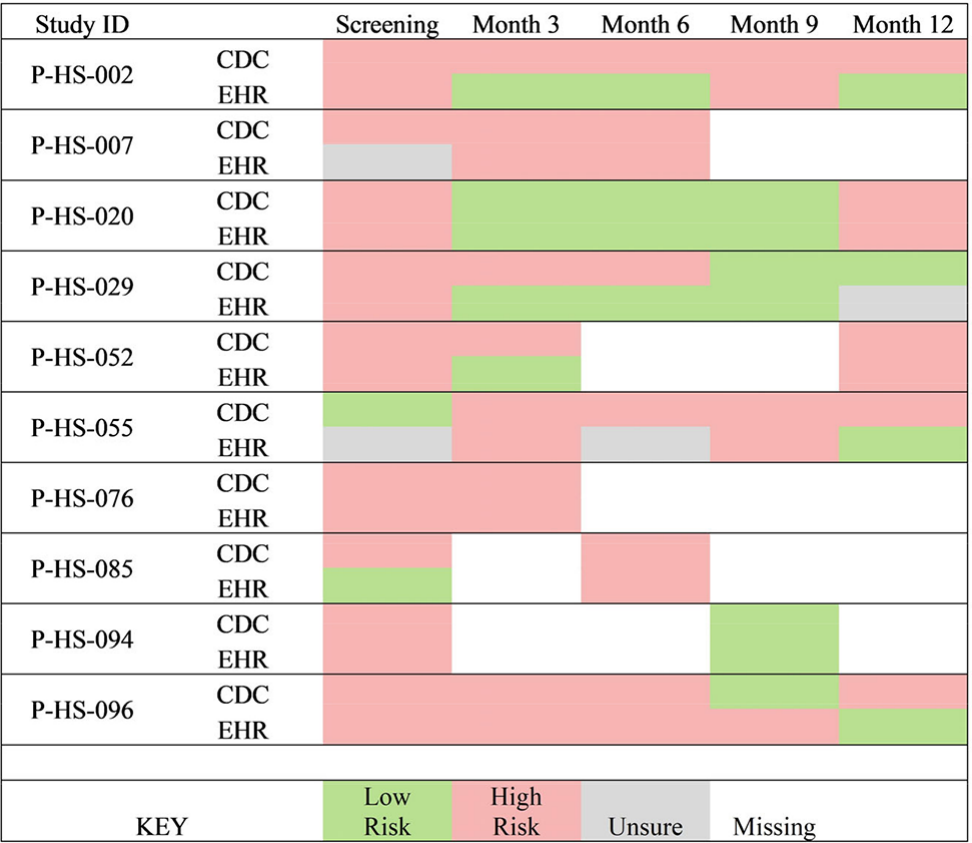
Fluctuating HIV Risk by CDC Guidelines and Endorsement of High-Risk (EHR) Among People Who Ever Endorsed High-Risk for Contracting HIV

**Results:**

Of 98 enrolled participants, 37 were HIV- and attended more than 1 visit, for a total of 151 visits.

At baseline, EE were more likely than NE to have been offered PrEP (100% vs 37%, p < 0.01), have taken PrEP (90% vs 22%, p < 0.01), identify as a trans-female (80% vs 33%, p = 0.02), endorse transactional sex (50% vs 15%, p = 0.04), and to have been diagnosed with a bacterial STI (70% vs 19%, p < 0.01). (Table 1)

Among EE, CDC PrEP eligibility and endorsement of HIV risk varied across time points and was discrepant. (Fig 1)

Across all timepoints, there was only a fair agreement between CDC recommendation to use PrEP and endorsed HIV risk (Kappa statistic of 34.2% 95% CI (19.1 – 49.2)).

At timepoints when participants met CDC criteria for PrEP, endorsing HIV risk was significantly associated with being on PrEP compared to those who did not endorse (72% vs 19%, p = 0.02). (Table 2) Among those not on PrEP or starting PrEP, 86% reported interest in taking PrEP in the future if they felt they were at risk for HIV.

**Conclusion:**

Among transgender participants, we found significant discordance between perceptions of HIV risk and PrEP eligibility based on CDC criteria, with self-perception of risk more significantly associated with PrEP engagement. Given high rates of willingness to engage in PrEP if they perceive HIV risk, better understanding this discrepancy and how to educate patients about HIV risk may be critical to improving PrEP uptake. Further, given frequently changing HIV risk based on CDC criteria and self-report, newer long-acting PrEP formulations may help to provide sustained protection against HIV in patients with fluctuating risk.

**Disclosures:**

Phyllis Bijole, BA, MA, GILEAD: HIPS had a grant from Gilead to perform community testing services that ended Janary 31, 2024. It paid a portion of my salary

